# Supplementary material for: The Role of Atmospheric Composition in Defining the Habitable Zone Limits and Supporting E. coli Growth
Source: Life (Basel). 2025 Jan 10;15(1):79. doi: 10.3390/life15010079 (PMC11766661; doi:10.3390/life15010079)
Supplement: Supplementary file 1 [file life-15-00079-s001.zip › Bottle_schematic.pdf]

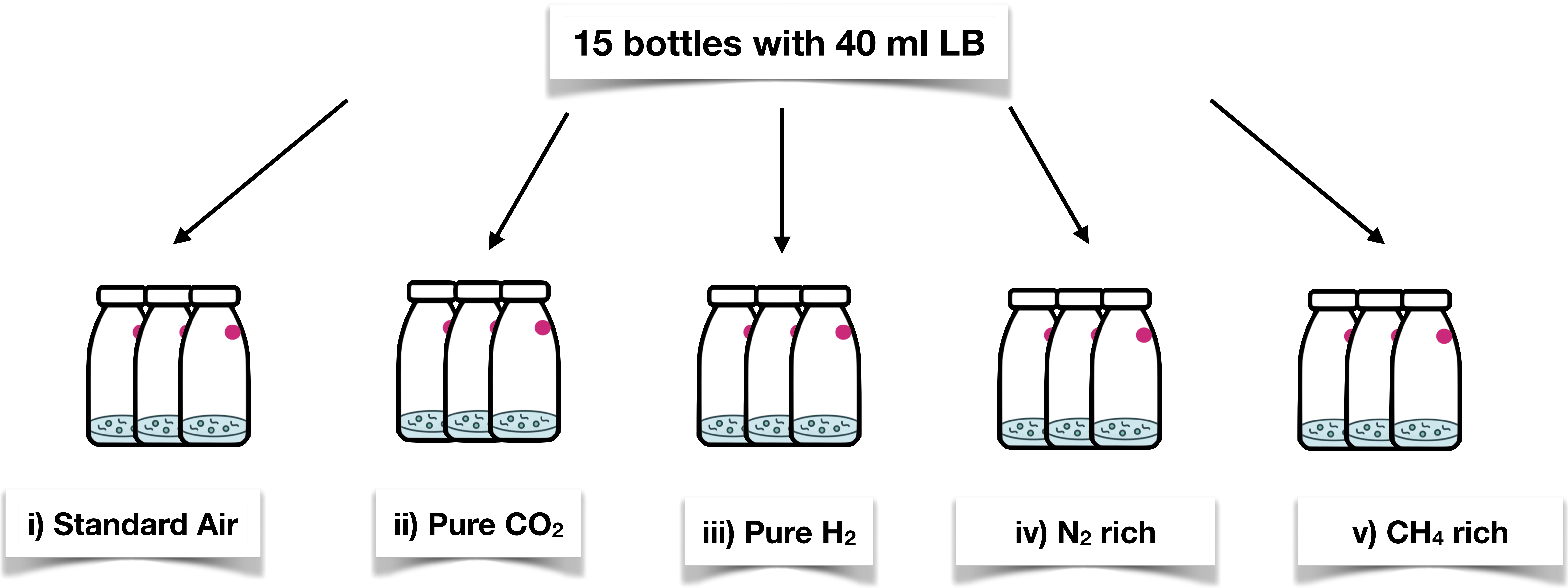

15 bottles with 40 ml LB
i) Standard Air
ii) Pure CO_2
iii) Pure H_2
iv) N_2 rich
v) CH_4 rich
