# Supplementary material for: The Role of Atmospheric Composition in Defining the Habitable Zone Limits and Supporting E. coli Growth
Source: Life (Basel). 2025 Jan 10;15(1):79. doi: 10.3390/life15010079 (PMC11766661; doi:10.3390/life15010079)
Supplement: Supplementary file 1 [file life-15-00079-s001.zip › HZ_limits_plot.pdf]

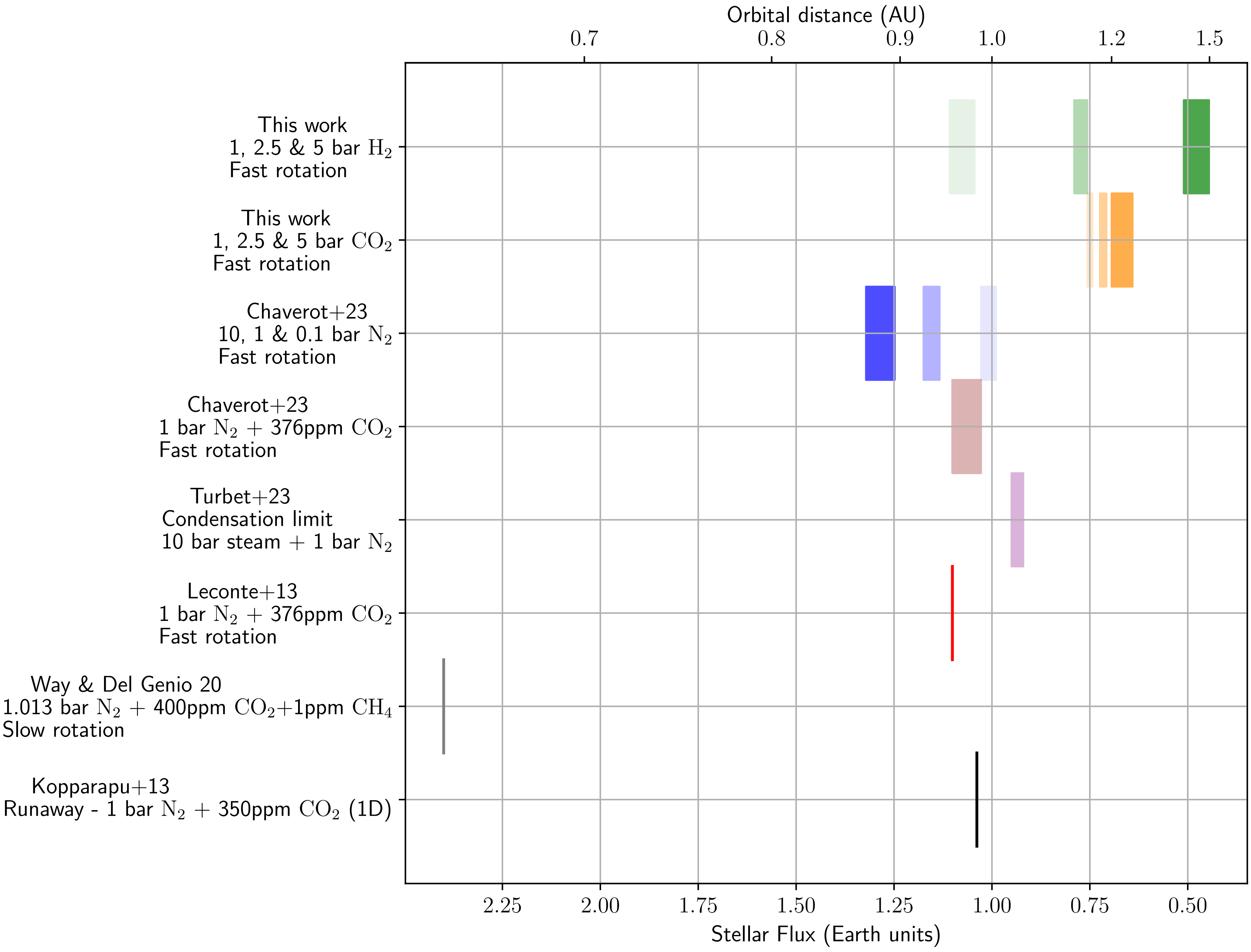

Orbital distance (AU)
0.7
0.8
0.9
1.0
1.2
1.5
This work
1, 2.5 & 5 bar H_2
Fast rotation
This work
1, 2.5 & 5 bar CO_2
Fast rotation
Chaverot+23
10, 1 & 0.1 bar N_2
Fast rotation
Chaverot+23
1 bar N_2 + 376ppm CO_2
Fast rotation
Turbet+23
Condensation limit
10 bar steam + 1 bar N_2
Leconte+13
1 bar N_2 + 376ppm CO_2
Fast rotation
Way & Del Genio 20
1.013 bar N_2 + 400ppm CO_2+1ppm CH_4
Slow rotation
Kopparapu+13
Runaway - 1 bar N_2 + 350ppm CO_2 (1D)
2.25
2.00
1.75
1.50
1.25
1.00
0.75
0.50
Stellar Flux (Earth units)
