# Supplementary material for: The Role of Atmospheric Composition in Defining the Habitable Zone Limits and Supporting E. coli Growth
Source: Life (Basel). 2025 Jan 10;15(1):79. doi: 10.3390/life15010079 (PMC11766661; doi:10.3390/life15010079)
Supplement: Supplementary file 1 [file life-15-00079-s001.zip › Supplementary.pdf]

Supplementary Materials: The Role of Atmospheric Composition in Defining the Habitable Zone Limits and Supporting *E. coli* Growth

Asena Kuzucan [1,2,*], Emeline Bolmont [1,2], Guillaume Chaverot [2,3], Jaqueline Quirino Ferreira [2,4], Bastiaan Willem Ibelings [2,4], Siddharth Bhatnagar [1,2,5] and Daniel Frank McGinnis [2,4]

This file includes:

Supplementary tables for Generic-PCM setup and supplementary image for experimental setup.

Table S1. Model parameters used for the simulations.

**Model parameters**	**Values**
Resolution: Longitude x Latitude x Vertical Levels*	60 x 48 x 30
Calculation of the dynamics (i.e. atmospheric transport)	Called every 96 s**
Calculation of the physics (evaporation, condensation, etc.)	Called every 8 min**
Radiative transfer	Called every 48 min**
Ocean model	2-layer ocean without dynamics
Ocean heat redistribution	false

* **Resolution specifies the number of grid cells in each direction of the 3D grid used in the GCM.**

** Numerical Time

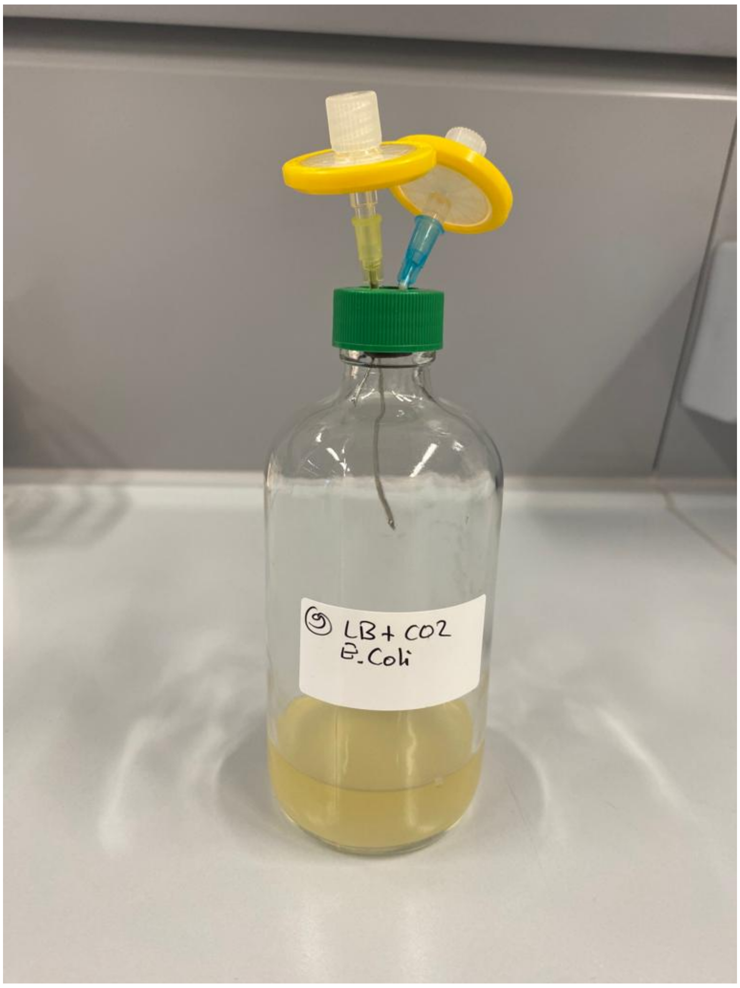

Figure S1. Photo of a bottle used for the pure CO_2 atmosphere experiment (ii). Like this bottle, each bottle has two needles with a 0.2 µm sterile filter for flushing with nitrogen and consequently filling the head-space with the desired atmosphere.
