# Supplementary figures and images for: The Role of Atmospheric Composition in Defining the Habitable Zone Limits and Supporting E. coli Growth
Source: Life (Basel). 2025 Jan 10;15(1):79. doi: 10.3390/life15010079 (PMC11766661; doi:10.3390/life15010079)

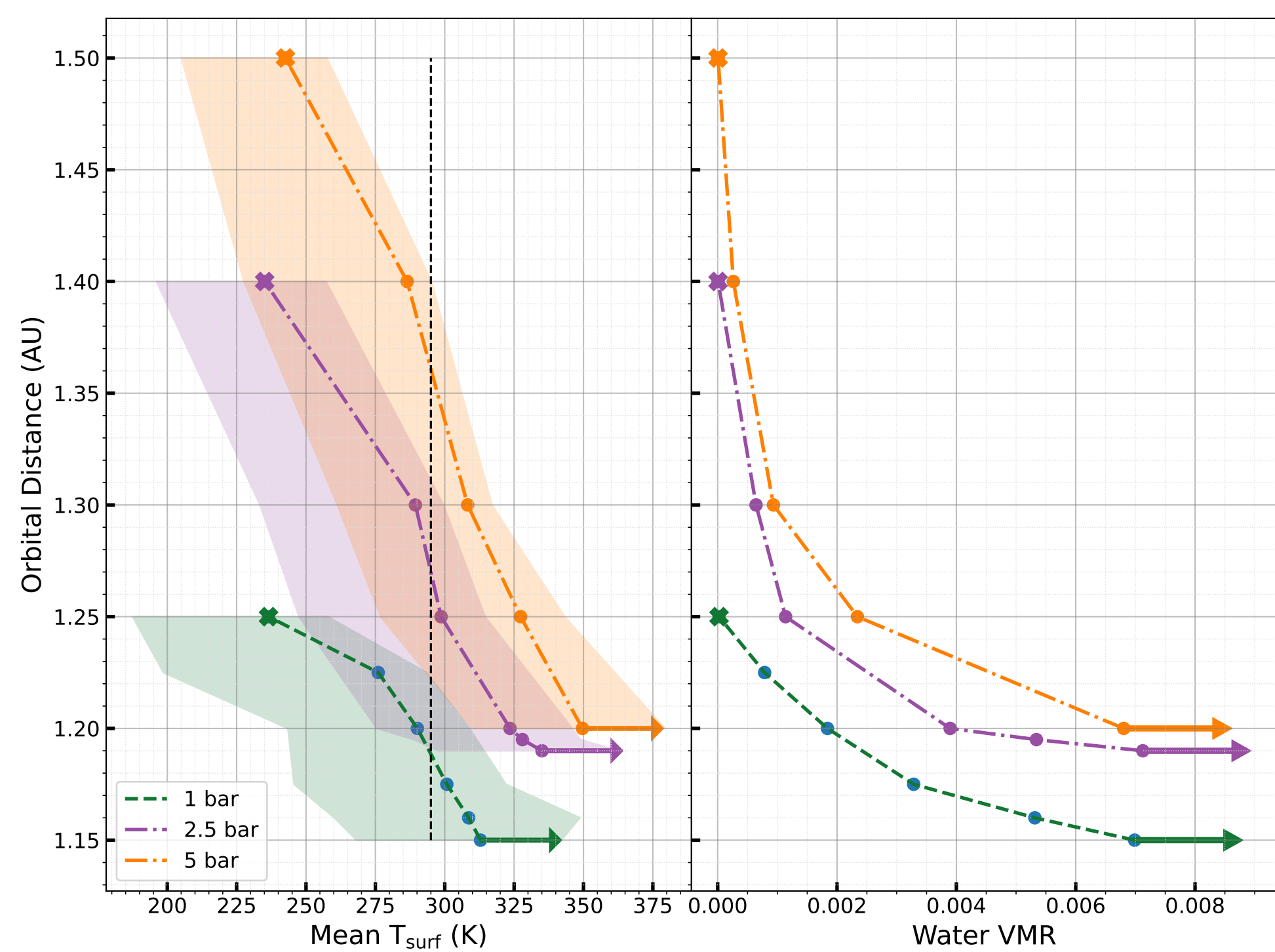

Supplement: Supplementary file 1 [file life-15-00079-s001.zip › CO2_Tsurf_vmr_vs_Distance.pdf]

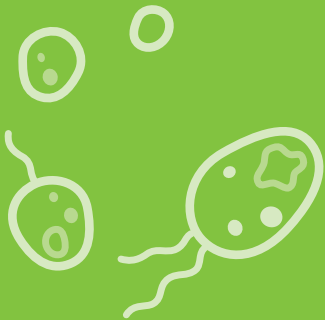

*life*

Supplement: Supplementary file 1 [file life-15-00079-s001.zip › Definitions/life-logo-eps-converted-to.pdf]

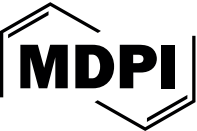

Supplement: Supplementary file 1 [file life-15-00079-s001.zip › Definitions/logo-mdpi-eps-converted-to.pdf]

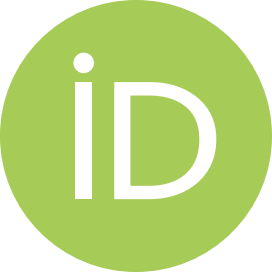

Supplement: Supplementary file 1 [file life-15-00079-s001.zip › Definitions/logo-orcid.pdf]

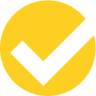

check for  
updates

Supplement: Supplementary file 1 [file life-15-00079-s001.zip › Definitions/logo-updates-eps-converted-to.pdf]

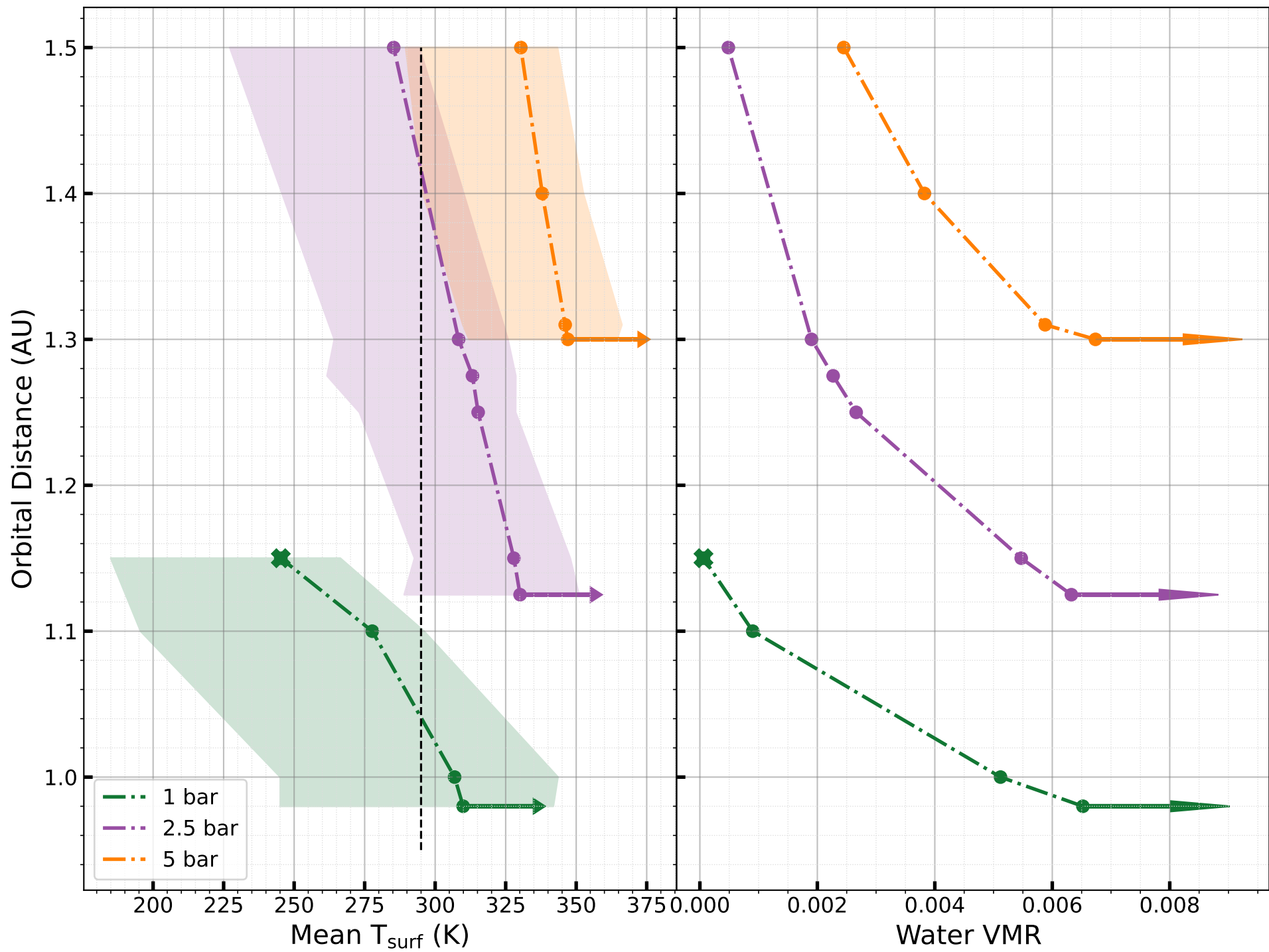

Supplement: Supplementary file 1 [file life-15-00079-s001.zip › H2_Tsurf_vmr_vs_Distance.pdf]

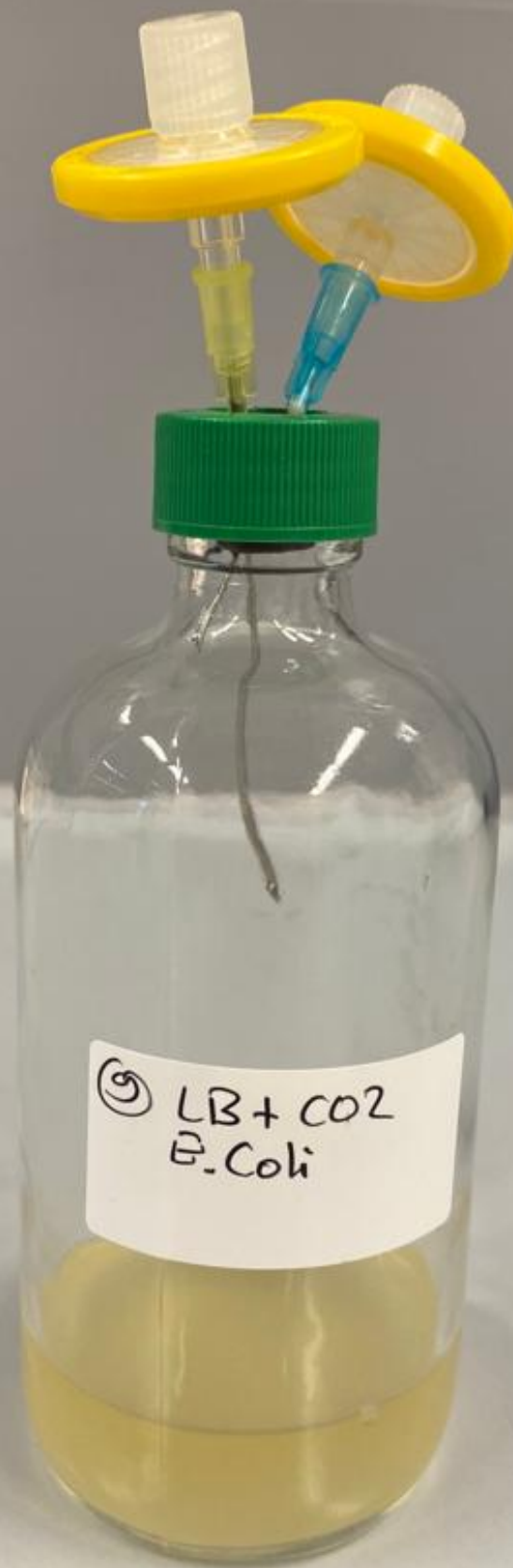

LB + CO<sub>2</sub>  
E. coli

Supplement: Supplementary file 1 [file life-15-00079-s001.zip › LB_with_CO2.pdf]

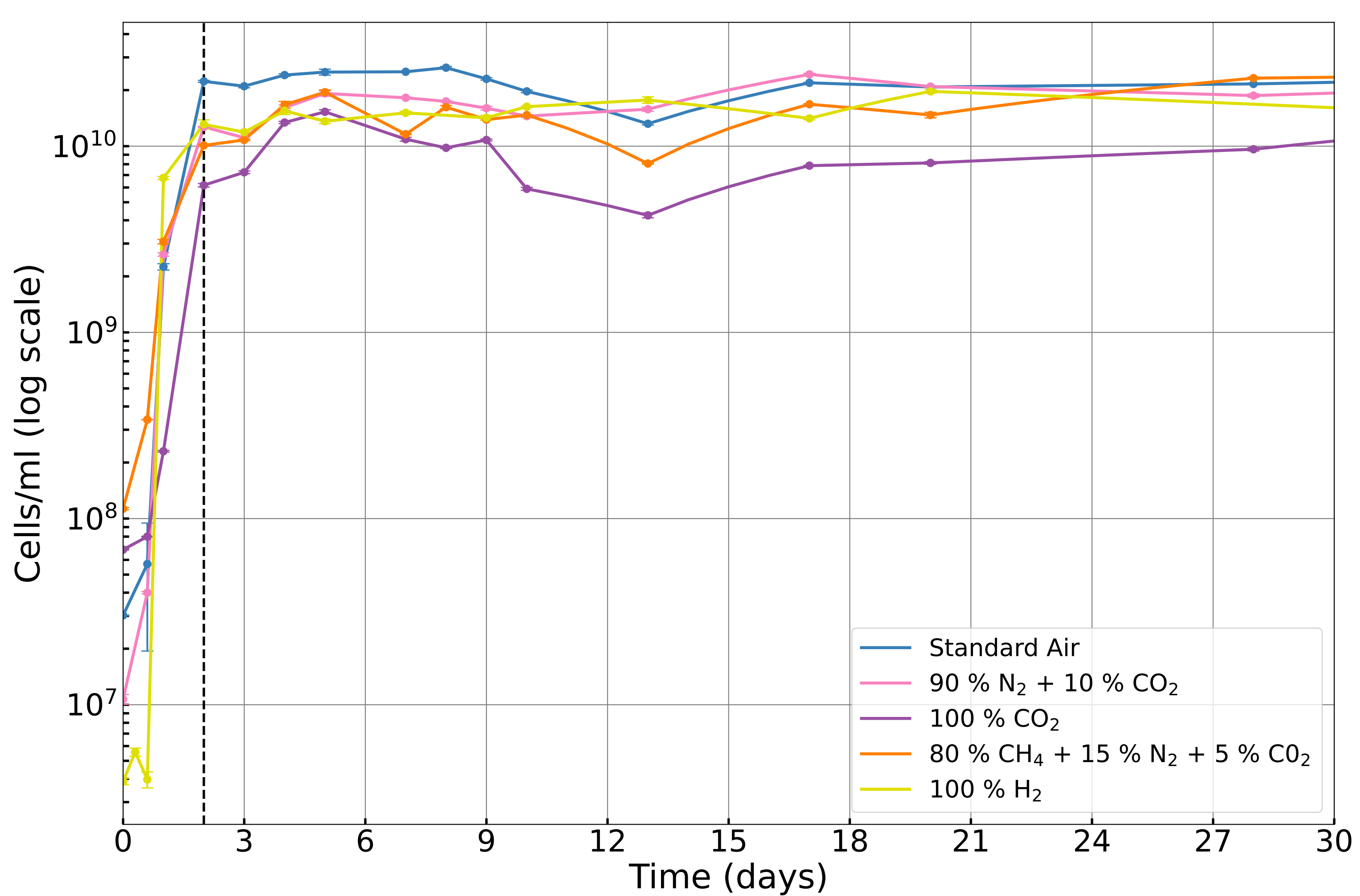

Supplement: Supplementary file 1 [file life-15-00079-s001.zip › Results_CASY_long.pdf]

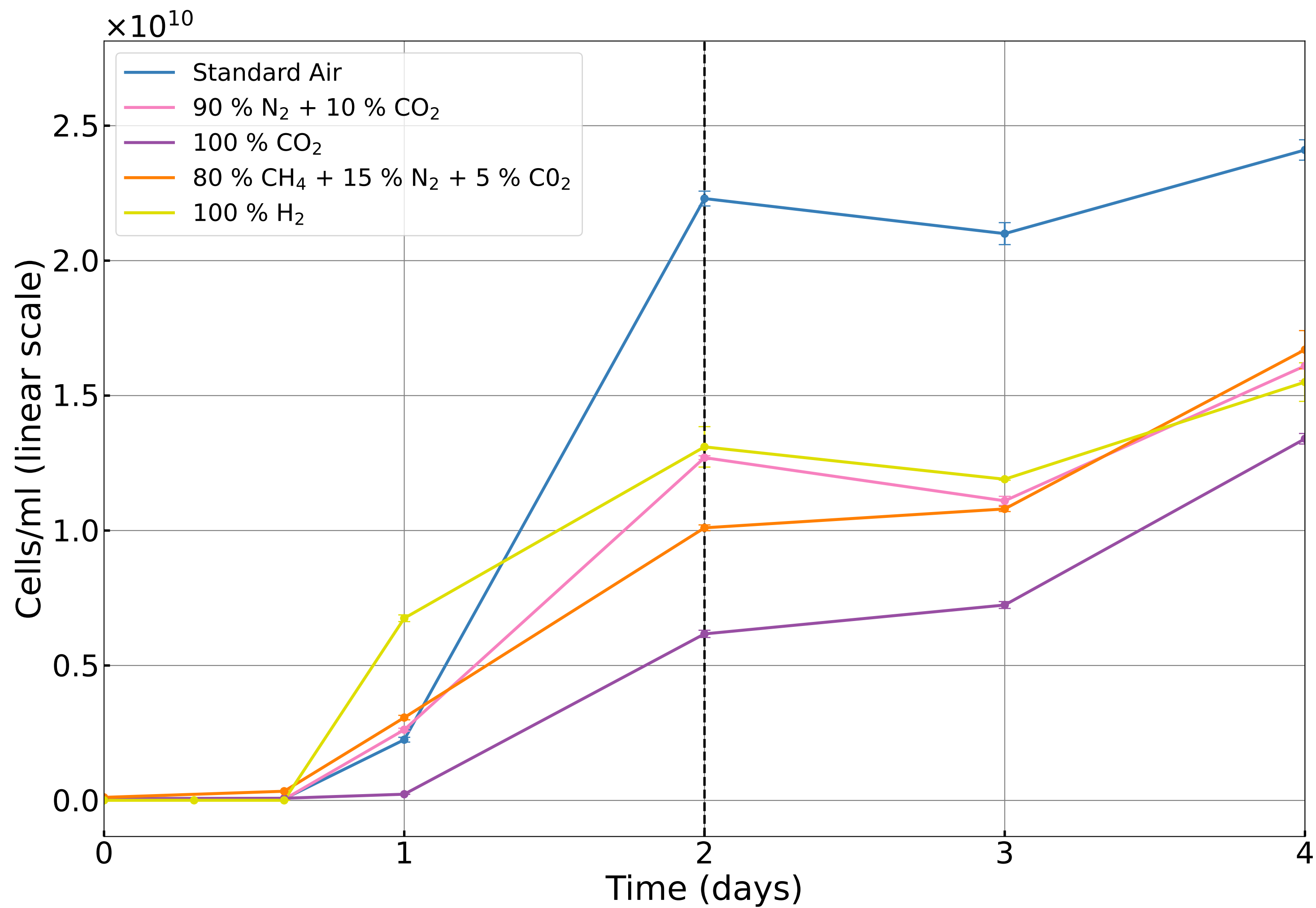

Supplement: Supplementary file 1 [file life-15-00079-s001.zip › Results_CASY_short.pdf]
